# Occupational nursing during disasters from the perspective of holistic theory

**DOI:** 10.47626/1679-4435-2021-910

**Published:** 2024-08-05

**Authors:** Beatriz Maria dos Santos Santiago Ribeiro

**Affiliations:** 1 Enfermagem, Universidade de São Paulo, Ribeirão Preto, SP, Brazil

**Keywords:** disasters, prevention and mitigation, nursing work, nursing theories, desastres, prevenção e mitigação, enfermagem do trabalho, teorias de enfermagem

## Abstract

Disasters are defined as unpredictable and complex situations. This theoretical-reflective study, conducted from August 2020 to December 2020, reflected on occupational nursing during disasters in light of Myra Levine’s holistic theory. The first principle of her model is to conserve human energy, focusing on vital sign measurement; the second, conservation of structural integrity, focuses on multiple injuries; the third, conservation of personal integrity, focuses on relationships; while the fourth, conservation of social integrity, involves public and private dimensions beyond an individual level. Levine’s conservation model is of special utility in disaster situations, since it is rooted in occupational nursing care, working toward effective adaptation of nursing processes for individuals and families, aiming to achieve professional autonomy and quality care for patients, their families, and society.

## Abstract (pt)

Os desastres caracterizam-se como situações imprevisíveis e complexas. Este artigo objetivou refletir sobre a ótica da enfermagem do trabalho em desastres, fundamentada na teoria holística de Myra Estrin Levine. Trata-se de um estudo teóricoreflexivo desenvolvido no período de agosto a dezembro de 2020. Na teoria holística, o primeiro princípio é a conservação da energia do ser humano, com foco na medida dos sinais vitais. Já a conservação da integridade estrutural foca nas experiências múltiplas com lesões. A conservação da integridade pessoal se refere aos relacionamentos para definirem a si mesmos. Por fim, a conservação da integridade social, por sua vez, envolve a definição do ser que vai além do indivíduo e possui dimensões pública e particular. O modelo holístico de Myra Estrin Levine contribui em especial com a temática de desastres, tendo em vista que, ancorada no cuidado de enfermagem no contexto ocupacional, baseia-se em trabalhar de forma didática a adaptação eficaz do processo de enfermagem aos indivíduos e às famílias, além de contribuir com o processo de enfermagem, visando alcançar a autonomia profissional e a boa qualidade da assistência prestada ao cliente, à família e à sociedade.

## INTRODUCTION

Disasters, defined as unpredictable and complex situations, require the work of several types of professionals, ranging from care provision for victims to the restoration of environmental and living conditions. Although disasters are more likely in certain locations, they can occur at any time or place.^1^ When faced with a disaster, nurses must be able to mobilize and solve problems, promptly meeting demands and providing relevant support through the available resources.

Nurses’ knowledge of disasters contributes to the organization and qualification of their work, especially in caring for affected victims. Nurses play a crucial role in care management, conducting workplace visits to critically and comprehensively analyze possible sources of accidents, as well as occupational diseases. Nurses have great potential to promote changes or improvements in these processes.^2^

In this context, occupational nursing is dedicated to ensuring comprehensive support for workers, the workplace, and occupational organizations. These professionals promote and protect health and well-being and work to prevent accident risk and occupational diseases.^3^

However, occupational nursing could benefit from further disaster training. This article will explore Myra Levine’s holistic theory, which is based on the human as a dynamic whole in constant interaction with a dynamic environment, especially regarding adaptation in response to emerging situations.^4^

## METHODS

This study was based on theoretical reflection, intentionally selecting materials related to disasters and nursing. Between August 2020 and December 2020, the following descriptors from the Health Sciences Descriptors (DeCS) and Medical Subject Headings (MeSH) databases were used to select studies: Disasters, Prevention and Mitigation, Occupational Nursing, Disasters, Disaster Planning, and Occupational Health Nursing.

The Latin American and Caribbean Literature in Health Sciences (LILACS), Medical Literature Search and Analysis Online System (MEDLINE), Web of Science, and Scopus databases were searched.

The following filters were applied to refine the search: original studies in English, Portuguese, or Spanish published between 2010 and 2020 whose full text was available.

The titles and abstracts of the search results were assessed, and non-relevant articles were excluded. Those investigating occupational nursing in disaster situations were read in full in light of Levine’s holistic theory.

## RESULTS AND DISCUSSION

### NURSING CARE IN DISASTERS AND EMERGENCY SITUATIONS

One survey analyzed nursing care for multiple victims in disaster and emergency situations, highlighting the need to restructure teams. This study found a lack of standard basic requirements for emergency response, such as the definition of tasks, roles, and responsibilities. These findings can guide interventions to prepare professionals for emergencies.^5^

A systematic review identified the importance of disaster preparedness among nurses, showing that previous disaster training and experience can make a significant difference. Another finding of this review was that nurses are insufficiently prepared and do not feel confident that they can effectively respond to disasters; in other words, nursing professionals must be better prepared to increase their effectiveness in such scenarios.^6^

Another study evaluated nurses’ knowledge of and preparation for disaster management, finding that they do not have the knowledge or skills necessary to respond to disasters.^7^ Although disasters are uncommon occurrences, the study found that many nurses do not feel confident about responding to disasters and that research into nursing practice during disasters is extremely necessary.^7^

Another study investigated management and organization during disasters, finding that nurses showed great familiarity with triage, but less familiarity with epidemiology and decision-making. Thus, ineffective response to disasters indicates inefficiencies in the current system. The study recommended more workshops, annual training courses, and activities based on the care team’s specific needs, as well as continuing education courses for nurses.^8^ If disasters occur, they must have the control and confidence to provide assistance.

A further aspect of disasters was described: environmental suffering, which is a particular form of social suffering due to pollution or environmental damage through specific factors. Such changes can interrupt an individual’s relationship with the environment, resulting in feelings of desperation, identity loss, and a state of suffering in which the victim can no longer distinguish between places or recognize them as part of their daily lives.^9^ During disasters, nurses are fundamental in transmitting important information and supporting the mental health of victims.

A study conducted in Hong Kong analyzed nurses’ level of perceived competency during disasters and its implications for curriculum development and public health.^10^ In agreement with another study, it was observed that nursing processes must be organized and responsible, while nurses, together with the multidisciplinary team, must be aware of all their skills to assist victims.^11^

### OCCUPATIONAL NURSING AND HOLISTIC THEORY IN DISASTER SCENARIOS

Levine’s holistic theory was selected for this study due to its didactic and easy-to-understand conservation model, which allows effective adaptation of nursing processes for disaster victims. However, at first glance, the theory is more closely associated with preventable disasters. In such disasters, occupational nurses must deal with family complaints, fear, and anxiety, ie, in addition to medical assistance, occupational nurses must become figures of trust, refuge, comfort, and help.^12^

The first principle of Levine’s model is the conservation of energy, ie, measuring vital signs to determine a victim’s safe activity level. The second principle, conservation of structural integrity, focuses on activities to restore lifelong structural integrity in those with multiple injuries. The third principle, conservation of personal integrity, helps maintain individuality through personal choices. Finally, conservation of social integrity acknowledges the community dimension of an individual’s life, involving the victim’s social support system to increase support.^4^

It should be mentioned that, even in disasters with multiple fatalities, occupational nurses can provide care to survivors and their families. This involves conserving energy by offering comprehensive care to workers affected by the disaster, determining the energy required to sustain their daily activities, and further informing and encouraging them through home visits and lectures. Nurses can also encourage good nutrition and sufficient hydration, provide emotional support, listen to complaints, and help workers feel more confident, thus maintaining psychological balance after an incident. They must also be able to refer victims to any necessary specialists.

Lost energy after an incident can be restored by health professionals through words of encouragement and acceptance. Since Levine’s theory considers care holistically, it facilitates the recovery of health and vitality to face daily challenges,^4^ especially after events such as disasters.

Regarding the conservation of structural integrity, nursing can help minimize suffering and prioritize healing. Structural integrity has to do with the frailty of victims after significant events like disasters.

Regarding the conservation of personal integrity, nurses must actively listen, treat victims humanely, transmit trust, help restore the victims’ self-esteem, identify responses for anxiety, provide safety information, and contribute to recovery from the situation, including any necessary adaptations. Personal integrity is the conservation or recovery of the worker’s identity and self-esteem, especially since, according to Levine, certain experiences can lead to depression.^4^

In conserving social integrity, nurses can help victims recover social relationships, provide emotional support, encourage exercise, inform victims about safety and their human rights, encourage changes in health status, help identify and overcome resolvable problems, discuss professional life (without wasting energy) and life expectancy, and provide support through home visits, consultations, phone calls, etc.

Conserving social integrity involves connecting workers with family, community, friends, and religion. Since these groups are present in the worker’s daily life and can provide further help, occupational health care must recognize and take advantage of their potential. Further theoretical and methodological references are needed for nursing in the commercial sector.

Occupational nurses must recognize workers as social beings, protecting their emotional balance in the midst of trauma. They can assist in rapid and safe adaptation, including therapeutic actions to support worker well-being. Since the theory encompasses environmental aspects of healing, in some cases, victims who face relocation can be encouraged to change jobs or work sector.

Nursing can help conserve the patient’s energy, altering the environment and holistically observing organic responses to the adaptation process. Levine highlights the need for comprehensive care that aims to balance conservation of energy and integrity. Nursing can help provide the necessary adjustments to conserve the wholeness of the individual.^4^

Social and environmental suffering can be high during disasters, since forced relocation entails environmental, social, and personal factors. Many victims lose their homes, churches, communities, social and political support, jobs, and family and social ties, being separated from childhood friends, relatives, and neighbors. Even after disasters, those who continue to reside in their region can be impoverished through loss of ancestry and collective memory.^13^

As stated above, in severe disasters families must often relocate to distant places. A study of Argentinian farmers found that they feel their land is an extension of their body and identity, a living space invested with intense subjective experience, work, social relationships, and struggles. When displaced, they reported feeling that they lost a part of themselves, which could clearly affect mental health.^14^

The impact of relocation on work, lifestyle, and mental health was shown in an international study on compulsorily displaced populations in China. Commuting was associated with higher rates of depression and illness.^15^ The same could be applied to displaced disaster victims.

Finally, although this study achieved its objectives, it involved certain limitations, such as the lack of studies on the topic. Nevertheless, it can contribute to the knowledge of nursing professionals who must work in disaster situations.

## CONCLUSIONS

In reflecting on disasters from the perspective of Levine’s theory, it must be pointed out that worker health cannot be neglected, potential risks in the work environment must be recognized, preventive actions and safety measures should be developed, and ethical responsibility should be considered. Furthermore, structural changes to provide more democratic and effective forms of social organization are needed to defend the basic rights to work and health. Healthy work environments should also be maintained, respecting the ethical responsibility to prevent disasters in so far as possible.

Since it didactically deals with effective adaptation of nursing processes, Levine’s holistic model facilitated analysis of occupational nursing in disaster situations. The model also contributes to nursing processes by focusing on professional autonomy and care quality for patients, their families, and society.
